# Research metrics of Australian eating disorders researchers

**DOI:** 10.1186/s40337-025-01239-5

**Published:** 2025-03-31

**Authors:** Felicia Reed, Maddy Lyon, Anita Raspovic, Peta Marks, Sarah Maguire, Leah Brennan, Claire J. Foldi

**Affiliations:** 1 Department of Physiology, Monash University, 26 Innovation Walk, Clayton, VIC 3800 Australia; 2 Biomedicine Discovery Institute, Monash University, 23 Innovation Walk, Clayton, 3800 Australia; 3 Australian Eating Disorders Research & Translation Centre (AEDRTC), Sydney, NSW Australia; 4 Charles Perkins Centre, Inside Out Institute, University of Sydney, Sydney, NSW Australia; 5 School of Psychology & Public Health, Latrobe University, 133 McKoy Street, Wodonga, VIC 3689 Australia

## Abstract

Building the research capacity and capability of Australia’s eating disorder (ED) research workforce has been identified as a key strategy to respond to the increasing prevalence of EDs. However, there is currently a limited understanding of the research strengths and scope of this workforce and this is a barrier to capacity building efforts and to evaluating the impacts of these efforts. This study sought to understand and summarise the current research metrics of the top 50 research experts in Australia identified through Expertscape. Publicly available publication, citation and funded research grants metrics, were extracted from Expertscape, Scopus, SciVal, Dimensions.ai and researcher profiles and summarised. The results indicate that Australian Feeding and Eating Disorder (FED) researchers are competitive internationally, and are spread across professional disciplines with the highest representation from psychology. Expertscape researcher rank was associated with higher numbers of publications in feeding and eating disorders overall, but not to total outputs, field-weighted citation impact (FWCI), or number of publications in top percentile journals. Publications were heavily focused on Anorexia Nervosa. Public grants awarded to the identified ED researchers in Australia over the past 10 years were largely National Health and Medical Research Council (NHMRC) project grant schemes, targeting innovative and creative research across any area of health and medical research. Cumulative dollars awarded over the 10-year period up to 2023 were approximately $23.9 million AUD, roughly 6 times less than that awarded to Schizophrenia research. These results summarise the current state of Australian FED research, comprised of a productive high performing research workforce limited by inadequate research funding.

**Supplementary Information:**

The online version contains supplementary material available at 10.1186/s40337-025-01239-5.

## Abstract (plain language summary)

Understanding of the scope and nature of eating disorders (EDs) research in Australia is limited and this is a barrier to enhancing the capacity and capability of the ED research workforce. This paper provides a snapshot of ED research expertise and funding in Australia over the 10 years from January 2013 to December 2023, to provide an understanding of the current ED research workforce, to inform workforce capacity building strategies, and to provide data that can be used to monitor change over time.

**Supplementary Information:**

The online version contains supplementary material available at 10.1186/s40337-025-01239-5.

## Background

Eating disorders (EDs) are highly prevalent mental illnesses marked by disturbances in thoughts and behaviours related to food, eating, body image, weight, and shape [1]. In 2023, more than 1.1 million Australians (4.45%) were living with an ED, with > 10% of individuals having had an eating disorder at some point in their life [2]. The prevalence of EDs is of growing concern, with global incidence proposed to have doubled between 2000 and 2018, a figure likely to be much higher when undiagnosed individuals are accounted for, as well as those who do not currently meet diagnostic criteria [3]. Despite their widespread prevalence and significant impacts, recovery rates remain consistently low [4–6]. This is true for mental health conditions more broadly, for which the best available treatments yield only modest response rates [7, 8]. One reason for this is that early intervention is integral to successful recovery [4, 9, 10], yet only 30% of people with an ED will receive treatment at all and of those that do, only 20% receive treatment that is empirically supported [11]. Moreover, the average delay from onset to treatment is more than 5 years [10] which may, in part, be attributed to the limited awareness of evidence-based practice by clinicians in combination with ineffective generation of practical and accessible solutions by researchers [12]. Effective translation of research into practice requires establishing a cohesive workforce, with a clear research focus based on key knowledge gaps, together with ongoing financial and systemic support and collaboration between academic, clinical and other stakeholders.

Consistent, diverse, and widespread capacity and capability building efforts are needed to develop a research workforce that is equipped to address the rising problem of EDs. This was identified as a key strategic priority outlined by the Australian Eating Disorders Research and Translation Strategy commissioned by the Australian Government Department of Health in 2021 [11]. By performing an evaluation of research productivity and impact of Australia’s top 50 experts in the field of feeding and eating disorders, we aim to provide valuable insights into the current landscape of the ED research workforce, highlighting areas where Australian researchers are performing well, as well as gaps in the research workforce that should be addressed. These findings will inform efforts to enhance research capacity and impact.

Publication and citation metrics are commonly used as an indication of scholarly productivity and impact, and are used to inform grant and funding distribution, and the perception and prestige of a research institution [13, 14]. There exist comprehensive and accessible databases such as Scopus, Google Scholar, and Dimensions, that provide an overview of metrics designed to capture scholarly productivity and impact, and allow evaluation of researcher contributions, collaborative networks, and funding and publication trends. Unfortunately, these tools are unable to capture non-traditional research outputs that contribute to both knowledge gain and service implementation and this is an important limitation to these metrics. They do however provide a means by which to effectively track traditional metrics within a given research field over time. This has recently been undertaken in the field of psychology more broadly in both Australia [15, 16] and the UK [17], as well as to define the impact of certain events (e.g. COVID) on the ED research workforce [18]. However, there has not yet been a directed effort to evaluate the metrics of the interdisciplinary ED research workforce in Australia. Such an appraisal is essential for informing, evaluating and tracking the impact of capacity building exercises aimed at developing research strength within the ED field. This study aims to provide a snapshot of Australian ED research metrics that can be used as a baseline (normative) dataset to track the success of capacity building strategies.

## Methods

### Identifying the top 50 experts

The online platform Expertscape was used to identify the top 50 Global and the top 50 Australian research experts (as defined by Expertscape) within the Expertscape field of “Feeding and Eating disorders”, as defined in the most recent database update that occurred on July 22nd, 2023 (https://expertscape.com; version wg22p3138). To determine rankings, Expertscape extracts PubMed data from the previous 10 years and assigns a score to each research article (considering year, type and journal) and to each author on the publication. It is important to note that PubMed covers the biomedical literature, which precludes some topics and may present an incomplete picture of the FED field. It is therefore best to think of this identification tool as “Biomedical Aspects of [Feeding and Eating Disorders]” as described in the limitations page of the website. This search yielded 1,429 eligible articles and a ranked list of associated experts. There were 10 researchers on the original list who had a publication track record with an Australian institution over the past 10 years, but who did not appear to be currently residing in Australia (based on online researcher profiles) or had never resided here but that held honorary or affiliate positions at Australian institutions. We therefore excluded from our list of experts, any researcher with less than 5 publications linked to an Australian institution over the period of 2013–2023. Data were limited to the top 50, due to the upper bound of access from Expertscape being restricted to 67 entries. We subsequently focused on this curated list of the top 50 Australian Experts (i.e. the top 50 remaining after exclusions) for the remainder of our analyses.

### Characterising the top 50 experts: qualifications, publications and funding

The top 50 researchers from the final list were used to define the current research workforce within Australia. Affiliated research institution(s) reported on Expertscape were confirmed with online researcher profiles. These included Google Scholar, LinkedIn, Open Researcher and Contributor ID (ORCID.org) and the staff profiles pages on the websites of experts’ affiliated institutions. The same profiles were used to determine place/s of work, location of employment, tertiary qualifications, and year of highest education completion. Qualifications and recently held appointments were used to determine professional classifications if not explicitly stated.

The abstract and citation database, Scopus, was used to extract standardised research metrics for each of the top 50 experts (https://www.scopus.com/). The Hirsch index (h-index), a measure of both productivity and citation impact, and the field-weighted citation impact (FWCI), which considers the citation influence of publications within specific research fields, were directly extracted from the Author Metrics section of each expert’s Scopus profile. The total number of articles published across all fields was recorded from Scopus and used to calculate the percentage of total publications which have been in the field of ‘Feeding and Eating Disorders’ (FED) in the past 10 years (taken from Expertscape). To better understand the relationship between career stage, years publishing in the field of EDs and research productivity, we examined the number of years since the highest level of study was completed (doctorate/PhD) in the top 50 experts. As a number of discipline groups contribute to the treatment of eating disorders, which leads to the development of research programs in these respective disciplines, we were interested in understanding if there were discipline-specific differences in productivity and impact among the top 50 experts. Based on these classifications, we examined two metrics of impact, namely h-index and FWCI. For context, an h-index of 20 after 20 years of research activity has previously been defined as a ‘successful’ researcher [19], meaning the majority of our sample meet or exceed these standards. However, h-index values, which increase over time even in the absence of new publications, naturally favour more senior researchers and can vary widely between fields [20]. In contrast, FWCI accounts for differences in publication and citation practices between fields. The eating disorder diagnoses and related concepts that the experts conduct research on was recorded across 9 MeSH terms: anorexia nervosa, avoidant-restrictive food intake disorder, bulimia nervosa, binge-eating disorder, obesity, food addiction, feeding and eating disorders of childhood, orthorexia nervosa and general EDs. Using the ‘Topics’ section of expert’s Scopus profiles as a guide, experts were manually assigned to as many or as few categories that their work addressed.

In an effort to understand the funding landscape for ED research in Australia over an equivalent 10 year period, we used grants data collected from the Dimensions.ai database (https://app.dimensions.ai) to examine the allocation of funding from national competitive grants schemes administered through Australian funding bodies. To evaluate ED-focused grants more specifically, we performed a more general search for Australian-administered grants allocated under the Research, Condition and Disease Category (RCDC) search code of “Anorexia OR Eating Disorders”, over the same time period as above. Grants data from funding bodies worldwide is provided directly by funders themselves or from public sources. Funding data often comes directly from the funder organisation, especially where funders are Dimensions partners (see link for data sources https://app.dimensions.ai/datasources). The terms used for limiting grant outcomes displayed were Country of Funder (Australia), Country/Territory (Australia), Funder (Australian Research Council, National Health and Medical Research Council, Medical Research Future Fund), Active Year (between 2013 and 2023), and RCDC (Anorexia OR Eating Disorders). For comparison of funding allocated to ED research with another mental health condition with lower prevalence, we used the same limiting terms as above but replaced the RCDC with “Schizophrenia”.

### Data analysis

**Fig. 1:**
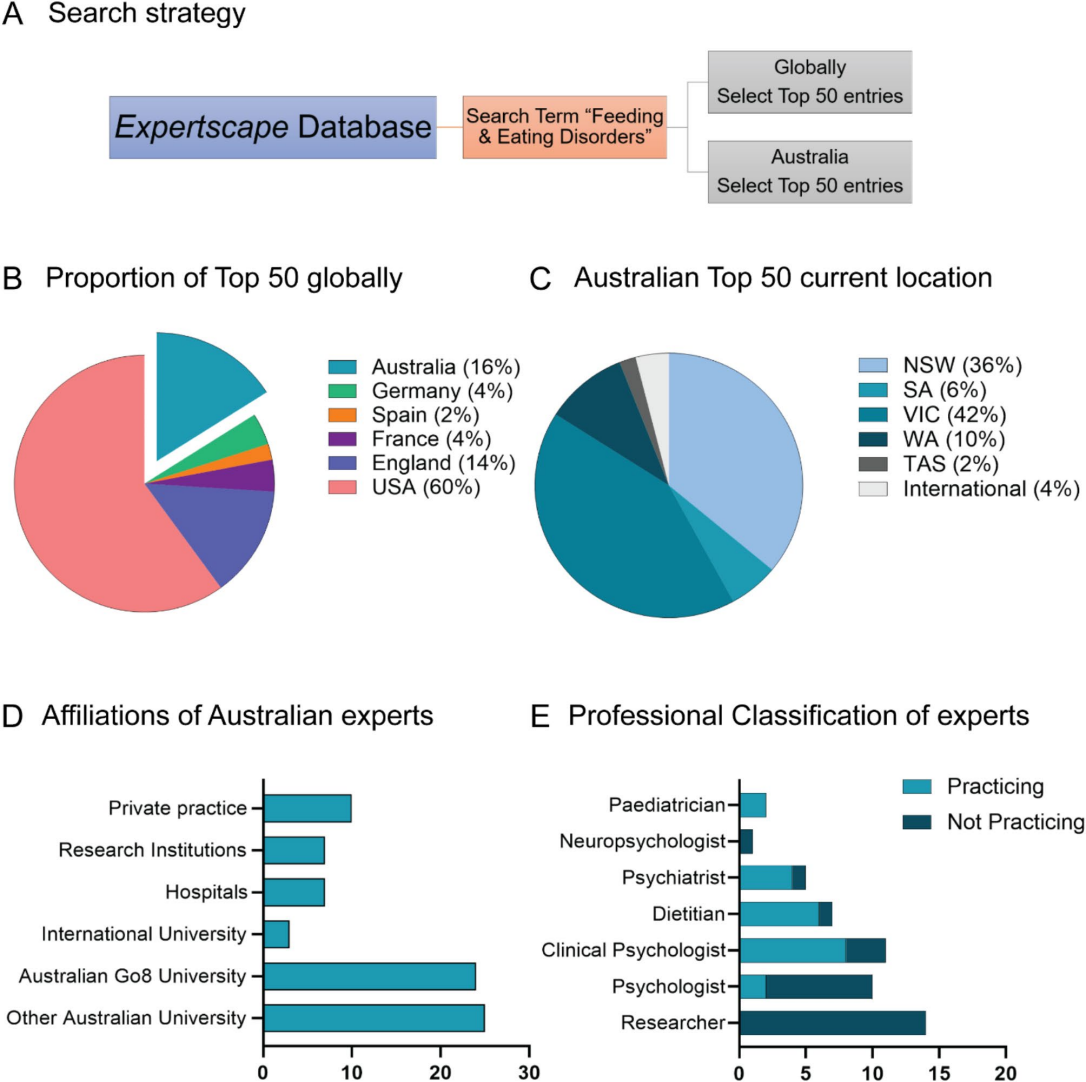
Characterising the top 50 Australian ED researchers. The Expertscape database search of the “Feeding and Eating Disorders” term revealed the Top 50 Eating Disorders (EDs) experts globally and those restricted to Australia (**A**). Australian researchers made up 16% of the global Top 50 (**B**), and were located predominantly at locations within Victoria (VIC) and New South Wales (NSW) (**C**). The affiliations of the top 50 Australian ED experts (more than one affiliation per researcher, where applicable) (**D**). Breakdown of top 50 Australian ED experts by professional classifications (qualifications), including experts currently practicing in their respective profession alongside research (**E**).

**Fig. 2:**
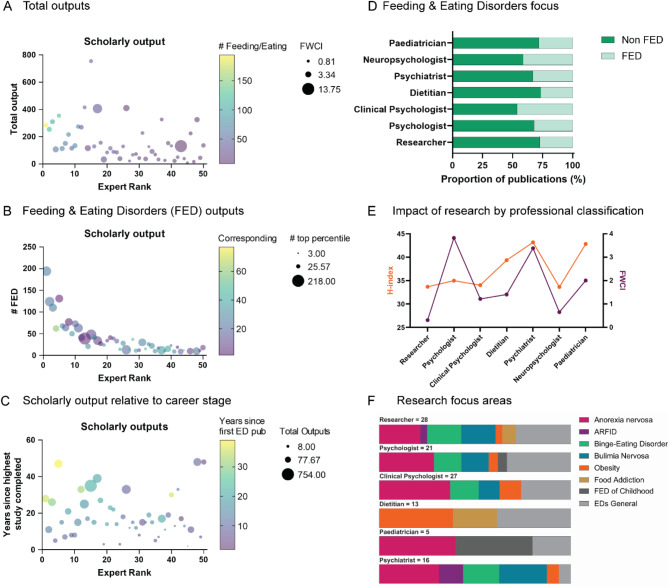
Australian top 50 Eating Disorders Researcher productivity and impact. Total scholarly outputs of Australian ED researchers by expert rank, with colour gradient representing the number of publications specifically feeding and eating disorders (FEDs)-focused (yellow is highest), and bubble size representing field-weighted citation impact (FWCI; larger is higher impact) (**A**). Number of FED-focused scholarly outputs by expert rank, with colour gradient representing the number of publications as corresponding author (yellow is highest), and bubble size representing number of documents in top percentiles of citations (larger is higher percentile) (**B**). Scholarly outputs expressed as number of years since highest degree was completed (e.g. PhD/doctorate) by expert rank, with colour gradient representing the number of years since each researcher first published in the field of eating disorders (yellow is highest), and total number of outputs for each researcher represented by bubble-size (larger is higher total outputs) (**C**). Proportion of total publications in the field of FED, by category of researcher training and professional qualifications (**D**). Impact of research by professional classification, using H-index (orange) and FWCI (purple) as measures (**E**). The distribution of research focus areas within ED research based on researcher professional classification (**F**). All scholarly outputs are related to the same time period (10 years up to 2023, inclusive).

Results were analysed with respect to Expert geographical location, affiliation and professional classification (Fig. 1). Scholarly outputs were assessed against Expert rank, career stage, impact and research focus (Fig. 2). Funding awarded was examined with respect to the amount and number of grants received for ED research. These were stratified by funding body, type of award, state in which funding was received and category of research funded. All collected (deidentified) data are available in the Supplementary Materials.

## Results

### Understanding the Australian ED ‘expert’– international standing, affiliations and professional classifications

Our search strategy identified the Top 50 Australian FED experts in the context of global research experts (Fig. 1A). Australian ED researchers had good representation (8 out of 50; 16%) and were second only to the US (30 out of 50; 60%). Other countries featuring in the top 50 international ED researchers were England (7 out of 50; 14%), France (2 out of 50; 4%), Germany (2 out of 50; 4%) and Spain (1 out of 50; 2%) (Fig. 1B). Of our exported list of Australian researchers (following the exclusions above), 7 out of 50 (14%) had expertise rankings within the top < 0.1% worldwide in the field, while 40 out of 50 (80%) had international expertise rankings within the top < 1% in the field (data extracted from Expertscape, not shown).

In order to understand where in Australia the bulk of ED research was being undertaken, we looked at the current location (State) and types of affiliations of the Australian top 50 experts (Fig. 1C and D). We found that the large majority of experts held positions within Victoria (VIC; 21 out 50; 42%) or New South Wales (NSW; 18 out of 50; 36%), consistent with these states being the most populous and having the most universities and research institutions. Of the remaining Australian states, 5 out of 50 experts were located in Western Australia (WA; 10%), 3 out of 50 were located in South Australia (SA; 6%), and 1 out of 50 was located in Tasmania (TAS; 2%). The following states and territories were not represented in the top 50 experts: Australian Capital Territory, Queensland, Northern Territory (data not shown). There were 2 out of 50 (4%) experts currently residing outside of Australia at international institutions, who had more than 5 publications with Australian affiliations over the past 10 years (Fig. 1C). We additionally classified researcher affiliations to understand representation across the research sector. All researchers had a minimum of one affiliation with an Australian university (52 total university affiliations). Of these, roughly half were affiliations were Australian Group of Eight (Go8)^1^ research-intensive universities (24/52), and roughly half were from Australian non-Go8 universities (25/52). The remaining university affiliations were international universities (3/52). Other affiliations included hospitals [7], research institutions [7] and private practice [10] (Fig. 1D).

Using information sourced from online researcher profiles, we classified the top 50 ED experts by professional qualifications, and the proportion of experts who were either currently or recently practicing alongside their research (Fig. 1E). Experts holding a higher degree by research with no identifiable clinical training (Researcher) featured most prominently in the top 50 (14 out of 50; 28%). Those trained in psychology were further separated into the following: Clinical Psychologist (11 total; 22% − 8 practicing, 3 non-practicing), general Psychologist (10 total; 20% − 2 practicing, 8 non-practicing), and Cognitive Neuropsychologist (1 total; 2% - non-practicing). Psychologists comprised 22 (44%) of the top 50 ED research experts. The remaining experts were either dietitians (7 total; 14% − 6 practicing, 1 non-practicing), psychiatrists (5 total; 10% − 4 practicing, 1 non-practicing), and paediatricians (2 total; 5% - both practicing).

Because both international collaboration and training is often seen as a valued attribute for researcher success [21], we additionally looked at whether any of the top 50 ED experts had completed any part of their training internationally. The majority of experts (62%) had completed all of their training within Australia, 20% had completed some form of training at an international institution, and in 18% of cases this information was not readily available online. Despite this, all Australian ED experts engaged in varying degrees of international collaboration (defined as the proportion of documents with an author with an affiliation outside of Australia), ranging between 6.1 and 84.6%, with an average of 39.8% of scholarly outputs including international collaborators.

### Productivity, impact and research focus of the top 50 ED research experts

Expertscape and Scopus were used to understand the relationship between expert rank and various measures of researcher productivity and impact. Consistent with the Expertscape ranking strategy, experts who ranked higher also had a greater number of publications in Feeding and Eating Disorders (FED), while experts ranked lower had fewer FED papers (Fig. 2A). While Expertscape rank was associated with number of FED related research outputs, we found no clear relationship between expert rank and total research (scholarly) output, (across all topics) and top-ranked experts did not consistently hold a higher mean field-weighted citation impact score (Fig. 2A). The absence of a relationship between expert rank and the number of total outputs as corresponding author, or documents in top percentile citations (Fig. 2B) suggests Expertscape rankings are not simply explained by seniority. Variation in the time since doctoral award was large within our expert group (Fig. 2C), ranging from 0 to 48 years (mean 18.1 years), and was not consistent with Expertscape rank. Similarly, entrance into the ED field (based on first FED publication) ranged from 2 to 39 years (mean 14.2 years), and was not directly related to Expert rank (despite researchers with a longstanding track-record in EDs being more likely to be ranked higher).

With respect to the research focus of the experts, only 10 out of the top 50 experts (20%) had > 50% of their publication outputs in the FED category over the past 10 years. Clinical psychology was the profession with the largest proportion of total publications within the FED field (Fig. 2D) followed by general psychologists and then researchers. Overall, the most impactful research by these metrics was conducted by psychiatrists, using both h-index and FWCI as indicators of impact (Fig. 2E). We demonstrate that Compared to research-only experts, psychologists showed higher impact based on FWCI, whereas h-index metrics were comparable. There was a heavy representation for anorexia nervosa across all categories besides dietitian, who disproportionately produced outputs related to obesity and food addiction. Unsurprisingly, the majority of FED of childhood focus area was produced by paediatricians (Fig. 2F). Similarly, despite the high prevalence of OSFED and UFED within Australia in particular, no researchers were identified to be studying these conditions specifically, however, this is likely a limitation of the database search methods used, in which these topics are not defined explicitly in Scopus or MeSH terms or not being designated as “primary” categorisations because the number of outputs are low compared to other conditions.

### Funding for Australian eating disorders research

**Fig. 3:**
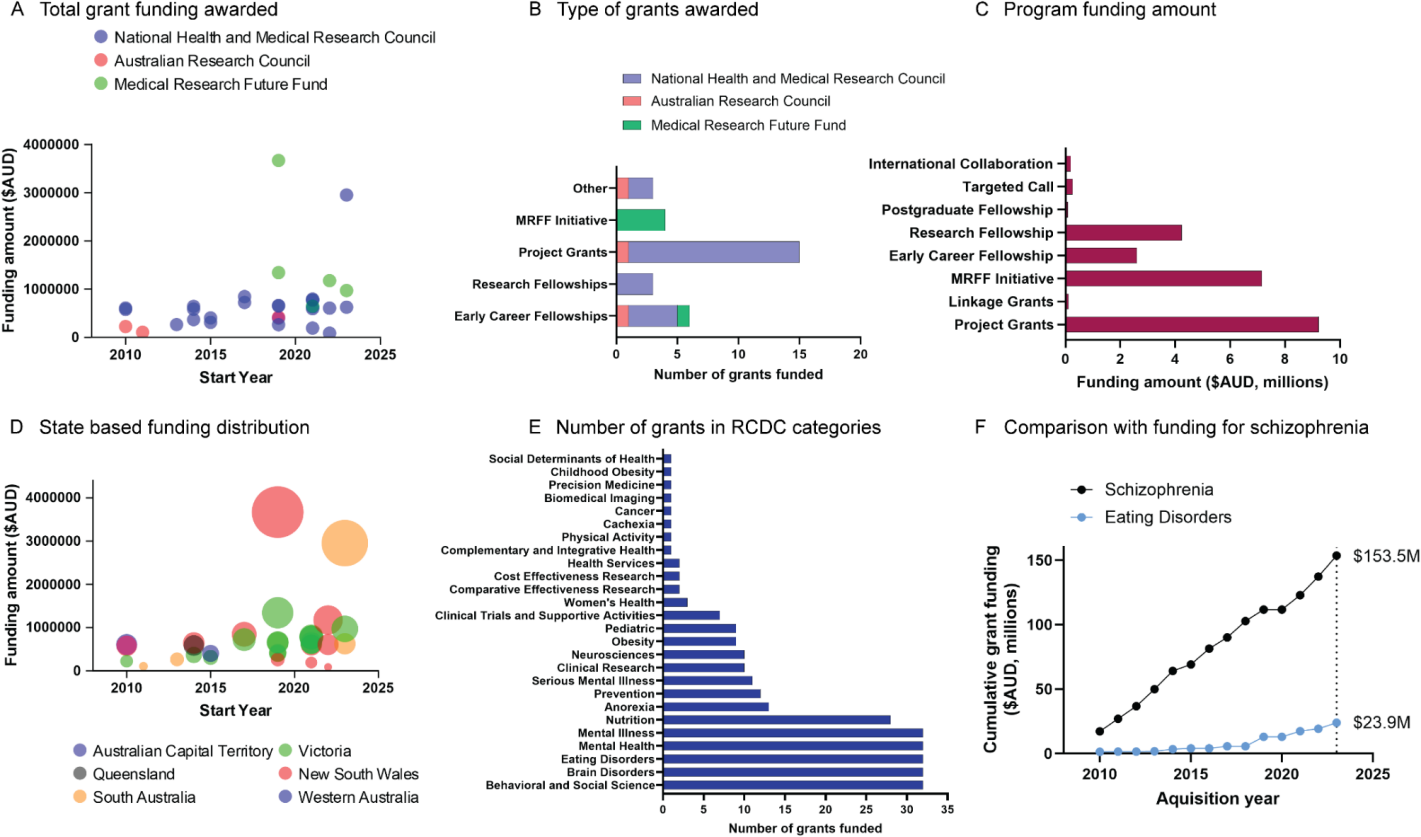
Funding for eating disorders research in Australia. Grant funding awarded over time to Australian research into eating disorders (EDs) by national grant funding bodies National Health and Medical Research Council (NHMRC; purple), Australian Research Council (ARC; pink), and the Medical Research Future Fund (MRFF; green) (**A**). The number of grants awarded by grant type and categorised by their associated funding body (**B**). The amount of funding (in $AUD millions) awarded to each type of funding program over the past 10 years (**C**). The distribution of funding across different Australian states and territories over the past 10 years (single bubbles represent single grants, and increasing size of bubble indicates increasing grant size in $AUD value) (**D**). Research, Condition, and Disease Categorisation (RCDC) assigned to each funded grant (each grant can have multiple) (**E**). The cumulative grant funding ($AUD, millions) allocated to ED research in comparison to a RCDC category with lower rates of prevalence in society, Schizophrenia (**F**).

Funding for eating disorders research comes primarily from the National Health and Medical Research Council (NHMRC– total 23 grants), with only 3 Australian Research Council (ARC) grants awarded over the last decade. The recent implementation of the Medical Research Future Fund (MRFF) in 2015 saw large-scale research funded (4 grants awarded), which did not increase over time (Fig. 3A). It’s important to note that in 2022, a $13 M federal grant was awarded to establish the Australian Eating Disorders Research and Translation Centre (AEDTRC), in response to an identified need to build research capacity in the field. This grant was omitted from our subsequent search strategy because it did not fall within the Dimensions search criteria, which identified a total of 32 grants awarded to ED research since 2013. These were primarily NHMRC project grants (including Ideas Grants– total 14 grants awarded) and 9 fellowships awarded at senior or early career levels (Fig. 3B). The number of funded grants aligns largely with the distribution of funds to programs, where we see the majority of dollars spent on project grants (>$9 M AUD) as well as the large-scale MRFF initiatives (>$7 M AUD; Fig. 3C). The distribution of funding across Australian states was heavily skewed toward NSW and VIC, as expected from the large proportion of researchers located in these regions, and was quite consistent across time (Fig. 3D). Similarly, the RCDC categories relating to mental health, eating disorders, brain and behaviour were over-represented in the grant funding awarded, with all 32 identified grants identifying the same 5 categories and with anorexia nervosa being the most common specific ED category identified in funded grant (Fig. 3E). For comparison, cumulative grant funding for EDs over the last decade reached 15.5% of dollars allocated to schizophrenia, with $23.9 M AUD for ED research compared to $153.3 M for schizophrenia research (Fig. 3F), in line with previous reports [22]. This is a particularly striking comparison considering that the population prevalence of schizophrenia is lower than EDs (~ 1%) and accounts for just over 7% of the burden of mental and substance use disorders [23], whereas ED accounts for approximately 15% of this global burden [24]. The other feature of this comparison worth highlighting is that although funding increased across time for both ED and schizophrenia research, the slope is much steeper for schizophrenia research, which suggests ED research continues to fall behind other major mental health fields.

## Discussion

This study aimed to understand the demographics and research productivity and funding outcomes of the current Australian ED workforce through a focused examination of the top 50 ED researchers, as identified using publicly available metrics databases. We used the previous 10 years (as at December 2023) of publication and citation metrics and allocation of government funding to EDs as a means to understand the productivity and competitiveness of the current ED research workforce. We sought to define focus areas of FED research, the overall competitiveness of Australian ED researchers, and distribution of resources across states, institutes, professional classifications, and disorder focus in order to track the efficacy of capacity building efforts into the future. This study highlights that the Australian ED research landscape is productive but constrained by funding opportunities. We likely have lessons to learn from fields like Intensive Care that have developed cohesive clinical trial and research networks to support the development of high-quality research proposals - as a field - to ensure increased success in national competitive research funding schemes.

### Australian ED researchers and international standing

Our findings suggest that Australian ED researchers are competitive on the international stage, with 16% featuring in the top 50 experts globally. This is noteworthy given the significantly lower population and lower overall funding allocated to research and development in Australia compared to other top-performing nations. Outside of the context of EDs, Australia had a contribution of ~ 1% to global research and development (R&D), which is substantially lower than contributions by Germany (~ 6%), France and the UK (~ 2–3% each). In line with expectations, the highest number of ED experts were from the US, which makes the highest contribution to global R&D (28%), however there were no ED experts in the global top 50 from a range of other countries, for example China which contribute ~ 22% of total research and development dollars [25]. This demonstrates that Australia’s ED productivity is high in proportion to other countries with larger populations and relative R&D spending.

### Australian ED researcher classification and training

We show that Australian ED research experts are diverse with respect to career positions and productivity metrics, although those with a psychological research focus were heavily represented in our list. These insights are valuable in making strategic decisions about potential workforce development activities and developing a baseline from which changes in workforce productivity can be tracked. For example, those who receive training in a narrow field (i.e. psychology, anorexia nervosa; see below) may be biased toward future research with an exclusive focus in that field. This could be avoided with more research support and training across disciplines to support a more diverse future ED research workforce. While 14 experts have a professional qualification as a psychologist, a further 15 experts with the professional classification of researcher have studied psychology at a tertiary level. Therefore, 29 (58%) of the 50 experts have studied psychology at any tertiary level, which is consistent with the prominent role of psychology in ED treatment and reflects the emphasis on research training within the psychology curriculum, with research conducted by students at the undergraduate, masters and higher degree research (HDR) levels. This also shows that if research is a core part of health professional training, it has very positive impacts on research outputs. While not all of those with a background in psychology will go on to conduct psychological research, it is valuable to understand that this is a common lens through which experts may approach their research.

The noteworthy observation that 75.61% of the experts completed their entire tertiary education within Australia suggests that the country’s education system is effectively nurturing and producing skilled scholars in the field of eating disorders, reflecting positively on the quality of education provided by Australian institutions. Although there is a lack of comparative data on the location of tertiary education in other workforces, it is conceivable that this 24.39% comprises experts who initially studied in their home country before emigrating or Australians seeking a global perspective and diverse experiences and some Australians who studied overseas and then returned. While the exact benefits of international collaboration are difficult to quantify, they may include access to diverse perspectives, resource sharing, exposure to alternative training methods and increased recognition. The geographical isolation faced by Australian researchers may also have a role to play in funding competitiveness, which could benefit from investment in scholarship in international locations.

### Australian ED research focus

The concentration of research on AN likely reflects its high mortality and presence in clinical settings [26], despite its comparatively low prevalence within ED diagnoses (4%; [2]). This may, in part, relate to complex medical and psychiatric risk associated with AN. In contrast, other EDs are less well understood and less recognised (particularly with respect to overt indications like low body weight) and therefore funding is disproportionately allocated. However, these EDs have higher prevalence and high burden of disease, and efforts need to be made to achieve parity in research focus. With this in mind, many publications address EDs more generally, often when exploring the role of body image or social media in the manifestation of EDs. Obesity is not considered an ED but it was included in the field of research list for experts identified in the FED category, presumably because of the high cross over of researchers studying obesity and EDs. Research on eating disorders and obesity within this group of experts perhaps intersects due to their shared connections with body image, nutrition, and mental health. In addition, there are bidirectional relationships between obesity and certain eating disorders (BED, BN) whereby higher weight leads to increased disordered eating and in combination with weight stigma, disordered eating can often lead to higher weight (including obesity) [27, 28]. The crossover in research recognizes the complex interplay between these conditions, exploring common risk factors, such as societal pressures on body image and psychological and biological factors influencing eating habits [29]. We recommend that the ED research workforce be better supported to diversify research that has traditionally been of narrower focus, across a range of areas that incorporate the increasing variety of affected individuals (body builders, neurodiverse, gender, sexuality, culture) and in light of increasing recognition of diverse diagnoses type (including atypical-AN, avoidant-restrictive food intake disorder and complex co-presentations) and interventions (psychological / biological / social / peer support etc.).

Australian and New Zealand Standard Research Classification Fields of Research and HRCS Research Activity Codes demonstrated a prevalence of research relating to individual care needs and psychological aspects of the conditions. This may simply reflect the concentration of psychological expertise in this group of experts. However, this also speaks to the dominance of psychological research in the ED field more broadly. Topic modelling analysis published in 2022 which found that animal studies of food intake had become one of the least researched areas of EDs by 2020, despite their potential in contributing to understanding the underlying neuropsychological and physiological influences and consequences of EDs [30].

### Limitations

Efforts to capture the entire ED research workforce within Australia is challenging because many researchers work in adjacent fields related to EDs such as body image, obesity, metabolic research, nutrition, or feeding behaviour more generally. The alternative of using a survey-based approach, while informative in its own right, it would rely on researcher response and thus result in incomplete data. In addition, only the top 50 Expertscape-identified researchers were used in this study as a sample of the Australian ED research workforce. The use of this sample offers several advantages and limitations. The small, ED-focused group meant that researchers whose primary research focus in other fields were limited, thus greater specificity of results were maintained. Examining common features of the most successful researchers can tell us what is going well in the workforce and helps us identify common features that may have contributed to success.

It is important to acknowledge the limitations in the way in which Expertscape identifies individuals, including a lack of transparency of how publications are assigned to a specific MeSH term at the exclusion of other terms that may be equally relevant for the field. The reliance on online profiles for data collection introduces the possibility of incomplete or outdated information and there are often inconsistencies across publication databases, despite best efforts. Thus, the analysis presented may not fully capture the nuanced contributions to ED research of experts, such as their impact on policy, clinical practice, or the community. Moreover, as noted above, it relies on assessment of traditional publication and citation metrics, which by nature exclude important contributions made by non-traditional research outputs, particularly the clinical and lived experience workforce. The incorporation of clinical and lived experience perspectives and contributions to the entire research pipeline, from project conception and design through to publication and information dissemination, has become a priority for undertaking meaningful research that is likely to yield the most impactful outcomes for the intended recipients (consumers) of academic research [31]. While the impact of incorporating lived experience expertise into the ED research pipeline was not explicitly studied in the current article, future efforts should incorporate non-traditional research outputs and design ways to measure and track the contribution and impact of these outputs in a standardised way.

### Recommendations and future directions

Firstly, it is important moving forward that the research workforce engage with clinicians, service providers and lived experience experts to improve the quality of patient care and outcomes [32], which requires training, funding and time. Potential strategies for engaging health care providers in research training have been identified [33], but there is limited evidence that these translate to increased research capacity [34]. This highlights a requirement to move beyond observational studies to more rigorous quantitative intervention studies aimed at increasing research engagement and capacity. With respect to funding, diversifying the sources of funding for ED research, including identifying philanthropic and other non-governmental sources could be achieved through improved accessibility of information about funding opportunities as well as including grant writing training, support and mentoring embedded within curricula and research institutions. Considering that AN represents a small proportion of ED diagnoses within Australia, there is also a need to expand research focus across FED more broadly.

Given the small but productive ED research workforce in Australia, capacity building efforts should focus on the retention and upskilling of trainees from these world leaders. We would therefore recommend targeted strategies to integrate ED research into broader university curricula and mental health research programs. Building capacity in the eating disorders workforce requires tailored strategies at each level of the research continuum, ensuring that the needs of research users, contributors, and leaders are addressed systematically [35]. For research users (e.g. those with a lived experience, clinicians, policymakers and advocacy groups), the translation of knowledge could be improved by the development of user-friendly guides, infographics and lay summaries of research findings for specific audiences. The ability to engage with researchers via collaborative online forums would also enable research users to provide feedback on priorities and key knowledge gaps. For research contributors that may incorporate involvement of individuals with lived experience expertise, community members as well as clinicians, service providers and more junior researchers, the goal of capacity building should be to ensure inclusive, ethical and impactful research outcomes. Diversity in the workforce is also critical for decreasing health disparities experienced by underrepresented populations [36]. Thus, the ED workforce should support the training, recruitment and retention of socially, linguistically, and culturally diverse researchers. Strategies to support research leaders should include increased opportunities for funding, leadership training and streamlined roles that may involve the reassignment of competing priorities (e.g. teaching and administration). As research leaders, we need to foster collaborative networks across the mental health sector, establish structured mentoring programs for the emerging workforce and advocate for targeted funding schemes to support innovative research that aligns with the needs of those with lived or living experience of an ED.

### Perspectives

There is a misconception of EDs as being on the one hand, not problematic enough to deserve dedicated funding allocation, and on the other as being impossible to treat. This presents a barrier to training opportunities, research funding and implementation of research outcomes. In this context, it is the opinion of our research team that the funding directed to a national centre for eating disorders within Australia (i.e. the AEDRTC) is an important first step in raising awareness of the problem of EDs. This has positioned us to more effectively bridge research disciplines that jointly acknowledge the necessity for better outcomes. Moreover, we believe that recruitment and retention of enthusiastic and talented researchers to the field depends on increased funding, a supportive research environment and advocates to speak publicly about the need to sustain a strong research workforce in EDs. We believe that with the right confluence of expertise and research directed at innovative solutions, better outcomes for people living with an ED and their families are achievable.

## Electronic supplementary material

Below is the link to the electronic supplementary material.

Supplementary Material 1

## Data Availability

Data is provided within the manuscript and supplementary information files.

## Footnotes

1 The Go8 universities comprises Australia’s leading research-intensive universities. The Go8 leads and influences the development and delivery of long-term sustainable higher education research and policy. Go8 Universities feature in the top 50 for every broad subject area in the QS world university subject rankings.
